# Emotional sharing in football audiences

**DOI:** 10.1080/00948705.2019.1613159

**Published:** 2019-05-15

**Authors:** Gerhard Thonhauser, Michael Wetzels

**Affiliations:** a Institute of Philosophy, Technical University of Darmstadt, Darmstadt, Germany; b Department of Sociology, Technical University of Berlin, Berlin, Germany

**Keywords:** Emotion, football, social collective, emotional sharing, philosophy, sociology

## Abstract

The negative aim of this paper is to identify shortcomings in received theories. First, we criticize approaching audiences, and large gatherings more general, in categories revolving around the notion of the crowd. Second, we show how leading paradigms in emotion research restrict research on the social-relational dynamics of emotions by reducing them to physiological processes like emotional contagion or to cognitive processes like social appraisal. Our positive aim is to offer an alternative proposal for conceptualizing emotional dynamics in audiences. First, we offer a notion of emotional sharing for studying the social-relational dynamics of emotions. Second, we propose a working concept of audience as a dynamic and dispersed social collective. Finally, we bring these elements together in the description of two scenes of jubilation.

Imagine when a goal is scored during a football match. What happens in the stadium? A cheering crowd, making noise, screaming and clapping in joy. Now imagine a second goal – this time it is the winning goal. The emotional intensity is at its limit, something often described as ‘collective effervescence’ (Durkheim 1915): an ecstatic crowd of people who can no longer control themselves, giving way to a wave of collective joy. This is the established view on audience emotions in the scientific literature, and one that can also be found in everyday talk about football. But is this really what happens in a stadium? Are collective emotions so powerful that people cannot help but surrender to the effervescent emotions of the crowd?

In this paper, we follow a double strategy: Our negative aim is to identify shortcomings in received theories that have hindered the development of adequate conceptual tools for researching emotional dynamics in audiences. With regard to audiences, we look into research on football audiences as well as the broader theorization of collectives. On both levels, we identify patterns of approaching football audiences, and large gatherings more general, in terms of politically charged categories revolving around the notion of the *crowd* (section 1). With regard to emotions, we discuss the dominant paradigms in 20th-century emotion research and show how they hindered investigations into the social-relational dynamics of emotions (section 2). Our positive aim is to offer an alternative proposal on how to conceptualize emotional dynamics in audiences guided by the idea that emotions always are enacted within specific *affective arrangements* and embody a specific *emotional repertoire* within a particular *emotional culture*. Thus, *emotional sharing* needs to be studied within these dynamic formations of socio-material settings, and affective relations governed by *feeling rules*. We begin by discussing what conditions need to be met for *emotional sharing* to be possible (section 3). Then we present a working concept of *audience* as a specific type of *social collective* (section 4). Having these understandings of audience and emotional sharing in place, we are in a position to offer a detailed description of what happens in the collective jubilation after a goal (section 5). The paper ends with a short conclusion and outlook on future research (section 6).^1^

## Deconstructing collectives: football audiences and the study of the crowd

The study of football in the human and social science has always been part of an interdisciplinary discourse. Although there are many differences between disciplinary cultures, we find similar traits in various research traditions. More specifically, we submit that research on football audiences has been dominated by discourses centered around the concept of the *crowd*. We trace this dependence on crowd semantics back to the origins of sociology and show how it is still operative in contemporary social ontology.

### Research on football in Germany

It hardly needs an argument that the most popular sport in Europe is football (Krüger, Herzog, and Reinhart 2018). However, as research strategies differ from country to country and from football culture to football culture (Waine and Naglo 2014), we focus on the German research landscape, as this is also the context of the example we will discuss later on. Although there was some work on football and ideology already in the 1970s (Vinnai 1970), we can locate the beginning of scientific interest in football in the late 1980s.^2^ Authors like Heitmeyer and Peter (1988) characterized different types of fans in their case studies of violent football fans across various football clubs in Germany. In the years since, three main research lines can be distinguished. The first line focuses on ideal types of *fans*. Heitmeyer and Peter’s introduction of ideal types of fans set a starting point, which allowed other researchers to differentiate these ideal types into more specific social groups (e.g. ultras, hooligans, etc.) (Utz and Benke 1997). Moreover, groups like *ultras* have become a main subject in the field of fan research in Germany, focusing on political intentions (Gabler 2010), social interactions in stadiums (Winands 2015), and their organizational structure (Kathöfer and Kotthaus 2013). The second line focuses on *inequality* and *discrimination* in football. Although violence is still a prominent term in current research (Reichertz and Keysers 2018), it is nowadays more anchored in discourses of inequality and discrimination. Prominent research strategies focus on aspects like *sexism* (Sülzle 2011), *homophobia* (Degele 2013), and *racism* (Zifonun 2007). Looking at the third line, football is also part of studies of *mediatization* (Hebbel-Seeger, Horky, and Schulke 2016), especially in combination with *commercialization* (Schwier and Schauerte 2006). Clubs are no longer bound to their core business, playing football in their local sports systems. They transformed into international businesses (Hebbel-Seeger and Förster 2008), being sponsored by even bigger enterprises (Oppenhuisen and van Zoonen 2006). The mediatization of football is an important accelerator of its commercialization, as the popularity of football has become a worldwide phenomenon, with the attention being more and more on players, coaches, and the mediatized, dramaturgical structures of football (Schwier and Schauerte 2009).

What we identify as potential problems of the described research lines is their fixation on social groups like ultras, mostly in combination with negatively connotated issues like inequality or violence. Although we consider it important to talk about these issues, we think that the research field is restricting itself here. By only talking about specific social groups and their ‘negative’ public image, we are restricting the empirical research to specific issues and losing sight of many important social dynamics in football audiences.

### Football, sports and the discourse of crowds

Our suggestion is that this fixation can be explained with reference to its historical lineage. In order to understand the case of football research, we first need to consider the location of sport more generally within the social sciences. We again restrict ourselves to the German research landscape. For a long time, sport was not a popular topic of research, as it was considered as bodily-offensive, undemanding and filthy. Even in the few texts that were published, sport was not seen as a positive influence, but as a phenomenon of losing people from intellectual leadership to the filthiness of crowds (Risse 1981; Krafft 1925). Consequently, sport as research subject was doomed from the beginning. Never entirely getting rid of the negative image of filthiness, it ‘is often underpinned by either an almost evangelical fascination […], or an intense critique of sport as an institution rife with social problems […]’ (Atkinson 2015, 8). Relating this burdensome heritage to current German football research, we can understand that the problems of sport are also the problems of football: Being torn between the extremes of fascination and horror, football audiences were never ‘just’ a research item, but always a politically laden issue, revolving around the notion of the crowd.

We cannot address the complex history of crowd semantics here (Borch 2012), so we restrict ourselves to pointing out its main aspects as they relate to the issue of this paper. The focus on negatively connotated social groups like ultras is not an isolated phenomenon, but part of a broader pattern which can be identified throughout the human and social sciences and which dates back to the origins of *crowd psychology* in the late nineteenth century. Led by psychological and behavioristic explanation models, authors like Scipio Sighele, Gabriel Tarde and, most popular, Gustave Le Bon described crowds as irrational, uncontrollable and inherently dangerous forces, symbols of terror and violence (König 1992). Especially in the theory of Le Bon, crowds were taken as psychological entities of their own – group minds – which were characterized by madness, leading to a loss of responsibility, individuality, and rationality on the part of the involved individuals (Le Bon 1896).

This powerful definition also reached German sociology. Max Weber, for instance, quotes Le Bon in the context of his definition of social action in *Economy and Society*: ‘It is well known that the actions of the individual are strongly influenced by the mere fact that he is a member of a crowd confined within a limited space. Thus, the subject matter of studies of “crowd psychology”, such as those of Le Bon, will be called “action conditioned by crowds”’ (Weber 1978, 23). Although Weber recognized Le Bon’s analysis here, he does so for excluding crowds from the subject area of sociology. According to Weber, sociology is concerned with *social action. Action* is defined here as behavior to which the acting individual attaches a subjective meaning. *Social action* implies that this meaning takes the behavior of others into account. Weber makes it clear that *action conditioned by crowds* should not count as *social action* (ibid.). Furthermore, Weber combined this distinction of *social action* and *action conditioned by crowds* with a distinction between *rational* and *emotional*: ‘Action is rationally evident chiefly when we attain a completely clear intellectual grasp of the action-elements in their intended context of meaning. Empathic or appreciative accuracy is attained when, through sympathetic participation, we can adequately grasp the emotional context in which the action took place’ (Weber 1978, 5). Weber made a far-reaching decision here: By separating crowd behavior (understood as emotional/irrational) from action (understood as rational and meaningful), he set the stage for a long-standing history of excluding crowd phenomena from sociological interest based on their ‘lack’ of rationality and meaningfulness.

Similar tendencies are at play in one of the founding texts of contemporary philosophical debate on collective intentionality and social ontology. Margaret Gilbert’s seminal work *On Social Facts* (1992) refers to Weber, Simmel, and Durkheim in an attempt to clarify the basic meaning of the term ‘social’. While Gilbert holds against Weber’s reductive account that ‘there is an important and theoretically respectable sense in which collectivities can act’ (Gilbert 1989, 15), she agrees with Weber in her hesitation to attribute genuine sociality to crowds. Gilbert’s proposal is to explain sociality through her *plural subject* account: For people to do things together they need to express to each other willingness to be part of the *plural subject* performing the action. When individuals make the commitment to join a plural subject this creates certain rights and obligations (for instance, you cannot one-sidedly leave the agreement). For Gilbert, sociality arises from such commitment, and she appears to hold that such commitment is precisely what crowds lack.^3^

Going back to the case of sport, we are now in a position to understand why received accounts of people inside stadiums are limited. As sport was primarily understood in terms of crowd dynamics, it was not considered a relevant subject to be studied in mainstream sociology or social ontology. Instead, it was studied on the theoretical grounds established by crowd psychology. As discussed in the case of Risse (1981[1921]), sport was considered a paradigmatic example of the filthiness of the crowd, which turned a pure, rational system into an emotional, dangerous battlefield. The interesting thing here is that, taking again a step further into the case of football, this suspicion was not addressed against the players on the pitch, but against the ‘symbol’ of the crowd – the *audience*. Audiences invoked the ‘old’ explanatory pattern of crowds and researchers reproduced crowd semantics in their research subjects, framing groups like ultras a priori as dangerous social groups (Heitmeyer, Scherer, and Winands 2010).

## Deconstructing emotions: research on emotions in the 20th century

In a second step, we discuss the leading paradigms in 20th-century emotion research and show how they restrict research on the social-relational dimension of emotions by reducing it either to physiological processes like *emotional contagion*, or to cognitive processes like *social appraisal*. To explain why this is the case, we take a brief look at the two leading paradigms of the interdisciplinary field of emotion research: the *somatic feeling paradigm* and the *cognitivist appraisal paradigm*.

### Traditional emotion research

The original version of the somatic feeling paradigm was developed at the end of the nineteenth century by William James (1884) and Carl Lange (1885), and is thus referred to as the *James-Lange theory*. Updated versions are currently defended by, among others, António Damásio (1994) and Jesse Prinz (2004). The gist of these theories is that emotions are best understood as feelings that are caused by a set of bodily changes. For instance, when an organism perceives a danger, this sets off a number of physiological responses. When the organism becomes consciously aware of these responses, we can say that the organism experiences fear. As a result, the somatic feeling paradigm understands emotions as physiological forces that require causal explanations. The same can be said about evolutionary theories of emotions. Paul Ekman (1972), for example, claims that expressions of basic emotions are biologically determined and thus follow a universal grammar. These expressions are associated with *affect programs* (Griffith 1997), which are understood as complex response systems operating below the level of consciousness. These approaches have the important consequence that the social-relational dimension of emotions must also enter the picture on the level of physiological processes. Many important findings have been achieved within this paradigm: It allowed researchers – mostly in the field of social psychology – to identify mechanisms of convergence like facial mimicry (Blairy, Herrera, and Hess 1999; Dimberg, Thunberg, and Elmehed 2000) and the imitation of bodily postures (Chartrand and Bargh 1999; Heyes 2011). In emotion research such convergence has been discussed most prominently in terms of *emotional contagion*, which is defined as ‘the tendency to automatically mimic and synchronize expressions, vocalizations, postures, and movements with those of another person’s and, consequently, to converge emotionally’ (Hatfield, Cacioppo, and Rapson 1993, 96). By locating the relational dynamics of emotions in automatic processes below the level of conscious awareness, the somatic feeling paradigm shows surprising similarities to classic crowd psychology, which also suggested that crowds are formed by sub-conscious processes of contagion and imitation.

As a countermovement to this focus on physiological processes, other scholars aimed at reintroducing emotions as a relevant topic for philosophers and social scientist investigating rational action. They did so by emphasizing the cognitive nature of emotions. According to the *cognitivist appraisal paradigm*, meaningful, intentional states directed towards objects and events in the world form the core of emotions. Most cognitivists within philosophy defended a view that associated emotions closely with judgments (Solomon 1993; Nussbaum 2001). They see emotions as evaluative judgments that are accompanied by an action tendency and a feeling component. Within psychological discourse, this is discussed in terms of cognitive appraisals (Frijda, Kuipers and Schure 1989; Scherer 2005). The main idea is that for an emotion to occur, a stimulus is appraised according to a range of cognitive criteria. It is this cognitive basis which allows one to individuate different types of emotions and which determines the other components of an emotion. It is thus not surprising that within this paradigm, the social-relational dimension of an emotion comes into play on the level of its cognitive basis. This is exemplified in the concept of *group-based emotions*, which is extensively researched in social psychology (Smith 1993; Smith, Seger, and Mackie 2007). The notion of group-based emotions combines the appraisal theory of emotion with self-categorization theory (Turner 1987). Self-categorization theory focuses on individuals seeing themselves as a member of a certain social group (e.g. fans of Hertha Berlin). When a self-categorization becomes salient for an individual, this evokes a particular social identity. The theory of group-based emotions suggests that a social identity can lead an individual to appraise a situation from the perspective of a group and experience emotions on behalf of that group. For example, because an individual self-identifies as a fan of Hertha Berlin, she appraises the scoring of a goal from the perspective of her social identity as a Hertha fan, and this leads her to break out into jubilation when Hertha Berlin scores a goal. In our view, this explanation shows surprising similarities to the safeguarding against crowd behavior identified above with reference to Weber’s foundation of sociology. In both cases, the conceptual determination of the relevant phenomena aims at restricting the field of investigation to processes of meaningful cognition, thereby leading to a neglect of affective and bodily dynamics.

### T*owards a new paradigm*

Our problem with both paradigms is twofold. First, both paradigms build on a problematic understanding of emotions. They fit most naturally with the assumption of a mind-body dualism, which makes it plausible to introduce clear-cut separations of cognitive and affective components. This assumption has come under serious pressure by current trends in emotion research claiming that emotions are characterized by an inextricable combination of rationality and emotionality, cognition and affect (Goldie 2000; Ratcliffe 2008). The alternative suggestion is that emotions are best understood as ‘felt evaluations’ (Helm 2001), meaningful orientations that are immediately felt. This *phenomenological turn in philosophy of emotions* fits well with new *trends in social science emotion research* which emphasize that it is impossible to draw a neat separation of cognitive (rational) and biological (emotional) processes (Röttger-Rössler and Markowitsch 2009). This new paradigm opened the path for a new understanding of emotions in terms of their relational and communicative nature. Emotions are not so much *in* the body as they are expressions *of* the body. More accurately, they are primarily located in the communication *between* bodies. It is thus not surprising that this new paradigm was accompanied by a new-found multidisciplinary interest in shared or collective emotions (von Scheve and Salmela 2014; Thonhauser 2018a).

This leads to our second reservation. Both paradigms fit badly with the concrete episode of shared affection and collective expression we encountered in our observations of football audiences – observations we will describe in the final section of this paper. The following sections will hopefully make clear that the social-relational dynamics in audiences cannot be fitted into either the paradigm of biologically determined processes of contagion and imitation, or into the individualistic and rationalistic paradigm of cognitive appraisals. In contrast to both paradigms, we suggest that we need a relational and dynamic understanding of emotions which goes beyond traditional dualisms of cognition and affect. Such an understanding needs to capture how emotions are at once rational and affective, cognitively meaningful and bodily felt. Moreover, it needs to consider how emotions are experienced and performed in social-relational settings and modulated by the specific emotional cultures of those settings. Because traditional research was content to describe audience emotions in terms of overarching concepts like the crowd, emotional contagion, group-identification or social appraisal, it has always ignored the concrete, local interactions in which an audience emerges and shows to others and to itself through episodes of emotional sharing that they ‘are’ an audience.

In order to reconstruct a proper understanding of the observed emotional dynamics in football, we have to take a step back and work on the basic concepts for approaching this phenomenon. In the next section, we will suggest an understanding of *emotional sharing* that allows one to capture the social-relational dynamics in football audiences. Afterwards, we will offer a reconceptualization of *audiences* as a specific type of *social collective*. Finally, we will bring these elements together in our description of two scenes of jubilation.

## Reconstructing emotions: approaching emotional sharing

We approach the issue of emotional sharing by discussing two conditions we consider necessary for emotional sharing to be possible, namely *diachronic* and *synchronic integration* (Krueger 2016; Thonhauser 2018b).

With *diachronic integration* we refer to the multidimensional shared background that enables a plurality of individuals to emotionally evaluate a situation in a similar way and to express corresponding emotions together. Most importantly, this background consists of *shared cultural knowledge*. In line with phenomenological sociology (Schutz 1974) and social constructivism (Berger and Luckmann 1966), we understand knowledge as *social form of meaning*, an embodied action that is related to its performance as ‘it is simultaneously perceived as being performed, so that conduct is always linked to a sensual meaning’ (Knoblauch 2013, 303). Moreover, knowledge has to be understood as a temporal phenomenon, based in the *sequential orders* of embodied action, acquired through processes of ‘sedimentation, routinization, and habitualization’ (ibid.). As for our empirical case, one needs to share ‘general cultural knowledge about the situation, such as the knowledge of football, football audiences or stadiums’ (Knoblauch, Wetzels, and Haken 2019, 167). Moreover, one needs to share knowledge about the specific modes of expression in the audience one is part of, such as team songs or cheers designed for the team. It has been suggested to call such an ensemble of repetitive, socially regulated forms of expressions an *emotional repertoire* (von Poser et al. 2019). Such a preconfiguration of appropriate emotional expressions has previously been studied under the term *feeling rules* (Hochschild 1979). For instance, one is expected to jubilate when the home team scores a goal, while it is forbidden to jubilate when the goal is scored by the other team. One will most likely experience rebukes and sanctions when violating these ‘rules’. However, there is also the option of not taking part in audience performances, pointed out by Goffman (1963) as *situational involvement*.

This shows that in order for individuals to join such collective cheering and chanting, they need not only *know* about the chants and songs, they also need to *care* about what is at stake in the specific situation and thus feel the urge to participate in the audience performances. In the terminology suggested by Bennett Helm (2001), we can say that the success of the team has *import* for the members of the respective audience. Something having import means that it is conceived of as worthy and valuable, as something to be supported and protected, promoted and developed.^4^ As members of the audience share such import, they also share the motivation to engage in coordinated actions in support of their team. As a consequence, the belonging to a certain audience is not only expressed by mere bodily co-presence, but also by taking part in supporting your team. Chanting and cheering, but also wearing a jersey of your home team are actions for making yourself recognizable for others and for yourself as being part of the specific audience.

Whereas research within the somatic feeling paradigm has mostly neglected diachronic integration, the integration of individuals into groups and their ability to experience group-based emotions has been an important focus of research within the cognitivist appraisal paradigm. However, this integration is usually accounted for in terms of group-identification, i.e. an individual’s self-identification as a member of a certain social group. According to our proposal, group-identification should be seen as a result of embodied knowledge and care and not as their basis. In other words, we suggest that group-identification and the corresponding social identity are the result of complex social processes leading to integration into a collective, and not their cause. For instance, the belonging to a specific kind of audience is not an effect of group-identification, but is preconfigured by the social and affective arrangement, embodied in specific forms of knowledge and care which have to be expressed through the participation in specific collective performances. By contrast, an exclusive focus on group-identification has the consequence that, in the end, it seems like it does not matter whether any interaction with other group members takes place, since an individual’s belonging to a group is taken to solely depend on her self-identification. An individual can imagine herself to be part of a group and experience emotions as if she were part of that group. However, this is not what is happening in a stadium, and likely also not the case in many other contexts.^5^

In contrast to the cognitivist appraisal paradigm, we suggest that diachronic integration alone is not sufficient to account for the specific episodes of emotional sharing that are part of such belonging. In addition, we need to account for the concrete interaction which enables coordination of the specific audience performances. We suggest calling this second aspect *synchronic integration*. Studying these interactional dynamics has been the strength of research within the somatic feeling paradigm. However, we are skeptical that such research is best conducted in experimental settings, as it is usually done within social psychology. Moreover, we disagree with assuming a universal grammar of emotional expression. By contrast, we consider it crucial to investigate in real-life contexts how members of a stadium audience are affectively resonating with each other, the players on the pitch as well as the socio-material setting, and how this is what constitutes their emotional experiences. Moreover, if we study these dynamics of affective resonance against the background of a social-relational and communicative understanding of emotion, it does not require us to separate the affective dynamics from rational (cognitive) attitudes and behavior. Rather, it enables us to understand *emotional sharing* as the collective experience of a gathering of individuals who share the import of a situation and interactively react to it.

In order to study the concrete emotional dynamics in a stadium, however, we also need an adequate understanding of the social collectives engaging in such emotional dynamics. Thus, the next section will discuss what makes an audience an audience.

## Reconstructing collectives: approaching audiences

As a point of departure for our reconstruction of *audiences*, we follow Christian von Scheve’s thoughts on *social collectives*. He offers a working concept of social collectives as assemblages of actors and objects that affect and are affected by others or by a specific situation (von Scheve 2019). According to this understanding, social collectives do not refer to some homogeneous unit, but to a plurality of individuals whose togetherness in a collective is bound to situation-specific circumstances. Only via being jointly affected by a specific situation, can individuals come to a self-understanding as part of a collective. The suggestion is that social collectives are ‘more effectively conceptualized and analyzed as *in the making* rather than as *substantial social formations*’ (von Scheve 2019, 267). They are not stable and encompassing entities, but transient and situational configurations.

Building on this understanding of social collectives, we can now suggest our working concept of *audiences*. Following Kolesch and Knoblauch (2019), we suggest an understanding of audiences along four key features:

(1) Audiences are a *specific type of collective* characterized by an assemblage of heterogeneous individuals sharing *a focus* on something. In philosophical terminology, we can say that audiences are in a situation of *joint attention* towards an *event*. In sociological terms, the suggestion is that audiences are entities coexisting with the development of an event, and more specifically, that audiences, as described in the case of knowledge, are *sequentially linked to* the specific *unfolding of an event* (Knoblauch 2016). For the purpose of this paper, we restrict ourselves to local audiences in which members are in bodily co-presence to each other and to the event (Knoblauch, Wetzels, and Haken 2019).

(2) Audiences are not just ‘passive’ receivers. By contrast, audiences can be characterized through a highly communicative, evaluative and energetic shared *activity*, which is often coordinated and prepared. In this context, it is important to note that an audience not only observes the unfolding event, but also what is going on within the audience itself. This enables members of the audiences, being in a situation of bodily co-presence, to mutually affect and be affected by each other. Moreover, it allows the audience as a whole to affect and be affected by the event as it is unfolding.

(3) Sharing a focus and being actively bound to the event and to each other, audiences are also defined by specific forms of *spatiality* and *temporality*. Through this feature, audiences can be identified as being located in certain forms of architecture and specific time settings. In our case, the audience we focus on is located in a prefigured social space, namely in a football stadium (Frank and Steets 2010). Football stadiums can be characterized as material settings with specific *social* arrangements (Goffman 1964). To provide an example: Certain institutional orders prevent supporters of the two teams from getting mixed up. This institutional setting ‘arranges’ the stadium audiences under a certain form of *agonality* (Knoblauch, Wetzels, and Haken 2019, 173). This agonal setting locates at least two audiences in different parts of the stadium, so that the mutual visibility does not only include one’s ‘own’ audience, but also the audience of the opposing team. Combining this third feature with the second, we can see that audiences are not just passively arranged in specific agential constellations. Rather, an audience actively engages in affective relations. Recently, Slaby, Mühlhoff, and Wüschner (2017) suggested the term *affective arrangement* to cover this complex assemblage which brings ‘[…] multiple human actors into a coordinated conjunction, so that these actors’ mutual affecting and being-affected is the central dimension of the arrangement’ (Slaby 2019, 109).

(4) Audiences are confronted with the *shared contingency* of a situation. Audiences face uncertain ‘futures’, as they do not know what will happen next. However, according to our proposal, it would be misguided to picture future situations as entirely unforeseeable. As part of a sequential order of embodied action, football matches follow recurring patterns. Having experienced these patterns in the past, people are able to recognize the kinds of future patterns to which a currently experienced situation might lead. Thus, an audience is usually in a good position to anticipate future situations, although there is always the possibility of another ‘future’. This anticipating suspense is characteristic of how audiences emotionally relate to the situational contingency of the event.

As we have discussed in section 1, large gatherings have not been a focal area of sociological research. This has been the case because sociology referred this issue to crowd psychology. Following the definition of Le Bon, crowd behavior was understood as driven by blind contagion and associated with irrationality, irresponsibility, and propensity towards violence. Understood in this manner, crowd behavior was sharply separated from social action, which was conceived as rational, individual action. Similarly, the philosophical debate on collective intentionality focuses on the cooperation of rational agents in small groups (Schweikard and Schmid 2013). All these approaches share the assumption that a sharp distinction can be drawn between rational action and irrational crowd behavior. With our proposal, we want to contribute to overcoming this impasse. Against an a priori distinction of rational action and crowd behavior, we suggest an empirically oriented approach that enables more nuanced conceptual work. Our hypothesis is that social collectives exhibit characteristics that can neither be reduced to rational actions executed by individuals nor adequately addressed in terms of crowd behavior. Instead, our working concept of audience is meant to enable researchers to take into account the particular interactive dynamics in specific socio-material settings – in our case, the dynamics of emotional sharing in football stadiums.

## Towards an account of emotional sharing in football audiences

Having developed these working concepts of *emotional sharing* and *audience*, we are now in a position to come back to the scenes described in the introduction, which is spatially and temporally located in the Olympic stadium of Berlin on 28 August 2016, during the first game of the new Bundesliga season between Hertha Berlin and Freiburg.^6^

**Figure 1.**
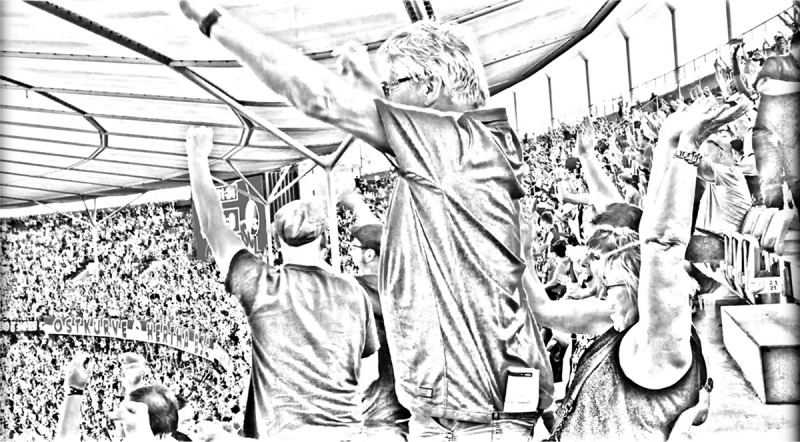
Celebrating the leading goal of Hertha Berlin against Freiburg in the 62th minute of the game, © Michael Wetzels.

The first scene took place in the 62nd minute, when Hertha Berlin scored their first goal of the season (see Figure 1). Let us take a closer look at the significance of that goal. First, in addition to being common knowledge that in a football game you need to score goals in order to win games, the audience members share the specific knowledge that it was Hertha Berlin’s first goal of the season. Moreover, it was the leading goal in the game. This is relevant because jubilation depends on the ‘affective dramaturgy’ of a game; for example, the ‘reaction’ to an equalizer (1–1) differs from the reaction to a leading goal (1–0) (Knoblauch, Wetzels, and Haken 2019, 172), as these events have different affective significance.

Second, already before jubilation broke out, we can observe that people were expecting a goal as part of the sequential order of the event. As mentioned above, the feature of shared contingency means that although people do not know when a goal will be scored, their experience with football games as they ‘typically’ unfold allows them to anticipate when a goal will likely be scored. Increasing noise and rising bodies make manifest this shared emotional knowledge (‘a goal might be scored’).

Third, we take these observations to be indicators of an active and performative audience. In raising their voices and bodies, people communicate that they know and care about what is happening on the pitch (diachronic integration), making themselves into an audience through being jointly affected by the evolving situation. In this context, the expressive behavior can be understood as a communicative act serving a coordination function (synchronic integration). In interaction with the focused situation, it enables emotional convergence among the audience. It is interesting to observe how this synchronic integration happens over time: As some people were already lifting up their bodies and began making noise, others were not yet involved. We could observe this with the help of video technology programs by looking at the exact time codes and the audio waves of the recorded situation. 0.7 s passed until everybody’s expectation were fulfilled and jubilation broke out among the members of the audience, lasting for 9 s. This shows how *becoming an audience* is an interactive and communicative process, in this specific case through the emotional sharing of jubilation.^7^

**Figure 2.**
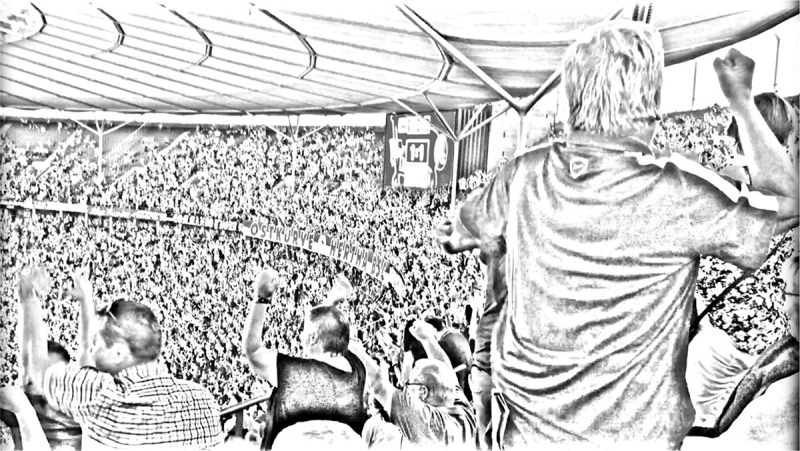
Celebrating the final goal of Hertha Berlin against Freiburg in the 95th minute of the game, © Michael Wetzels.

Let us now consider the second scene, taking place in the 95th minute of that game (see Figure 2). Again, we need to consider the specific ‘affective dramaturgy’. Just 3 min earlier, the away team scored the equalizer (1–1). As is common knowledge, a football game lasts for 90 min plus extra time. In this case, 5 min of extra time were displayed. The equalizer in the 92nd minute led to resignation of the supporters of Hertha Berlin. They knew that with only 3 min to be played, it was very unlikely for their team to shoot another goal for the win. However, this changed in the 94th minute. When the first goal happened in the 62nd minute, the length of increased noise before the goal was just 2 s. In contrast, the final goal was preluded by 15 s of increasing noise. It is part of the shared contingency of the situation that the audience knew that this may be the last chance to achieve victory, and thus, they were closely monitoring what was happening on the pitch. As the goal was scored, the different level of intensity compared to the first scored goal can be measured by how long jubilation lasted. While it lasted for 9 s following the first goal, it now lasted for over 1 min. This shows how cognitive and affective aspects of emotional sharing cannot be separated: Through the ‘logic’ of the temporal setting of a football game, the audience tacitly knew that in scoring a goal at this point of the game, their team could no longer lose – so they could rampantly express their collective joy.

To sum up, this in-depth description of collective jubilation shows that an episode of emotional sharing in a football audience happens in a social-relational dynamic setting. It is part of a meaningful and relevant emotional situation, which is related to the conditions of diachronic and synchronic integration and the key features of audiences (focus, activity, spatiality/temporality, shared contingency).

## Conclusion and future research perspectives

The negative aim of this paper was to show that traditional approaches to the research of audiences and emotions – especially approaches that revolve around the discourse of the crowd – are insufficient to capture the complex dynamics of emotional sharing in audiences. On the positive side, we advanced an understanding of audiences as dynamic and dispersed social collectives that are always in the making. Furthermore, we approached emotional sharing as a social-relational phenomenon that is always embedded in a specific socio-material setting, emphasizing the dimensions of diachronic and synchronic integration. Emotional sharing in audiences is an episodic phenomenon that is sequentially linked to the event, which is the audience’s shared focus. Moreover, it constitutively depends on the specific emotional culture and therefore only becomes comprehensible when seen against the background of the relevant cultural and embodied knowledge.

We would like to conclude this study with a few perspectives for future research: In terms of empirical research, a promising project would be a comparative study of the affective arrangement and the emotional repertoires in football with other forms of sport as well as with other types of cultural events (e.g. concerts, political rallies). For instance, it would be interesting to compare the exuberant emotional expressions in football with the disciplined audiences in tennis, or the defiant fan culture in football with the rather controlled and commercialized stadium experience in handball. In terms of conceptual work, an increased focus should be on modes of interaction in social collectives that are not defined by bodily co-presence, especially as processes of digitalization make sharp distinctions between face-to-face and mediatized interaction more and more obsolete. We submit that the approach to emotional sharing developed in this paper provides a generalizable framework for understanding emotional episodes in social collectives; a framework that enables empirical researchers to provide in-depth reconstructions of specific emotional cultures and their socio-material settings.

## Notes

1. The paper is co-authored by a philosopher with a background in existential phenomenology and a sociologist with a background in sociology of knowledge. While there is a link between our theoretical backgrounds via phenomenological sociology (Alfred Schutz), there are also theoretical and methodological differences that had to be negotiated in the research process – a negotiation we experienced as highly productive. Our work has been part of the Collaborative Research Center (CRC) 1171 *Affective Societies*, which develops an approach to the study of contemporary societies built on a social-relational and situated understanding of affect and emotion. Michael Wetzels’ work on this paper was part of the research activities of subproject C02 of the CRC. Gerhard Thonhauser’s work on this paper was part of a project associated with the CRC and funded by the Austrian Science Fund (FWF): J 4055-G24. We take this paper to show the merits of true interdisciplinary cooperation.

2. This interest was supposedly linked to the catastrophes in Hillsborough (Sheffield) and the Heysel stadium (Brussels), where many people died before and during a football game because of a so called ‘mass panic’.

3. It is difficult to determine exactly what Gilbert has to say about crowds, as she reports Weber’s distinction between crowd behavior and social action and then waives the issue of crowds as too complex to be further discussed in the context of her argument.

4. According to this theory, emotions can be seen as making manifest what is relevant and valuable to us. We develop this theory against the background of an understanding of affectivity (*Befindlichkeit*) as letting things appear as relevant, making us care, opening up a meaningful world in which things are experienced as valuable and worth caring about (Heidegger 1996, Sánchez Guerrero 2016).

5. We want to suggest that even a phenomenon like ‘basking in reflected glory’ (Cialdini and Bordon 1976) requires public appearance and visibility for others, and thus at least a minimal form of interaction, for example being seen by others as wearing the jersey of the team.

6. The analyses of these scenes were done through a hermeneutical understanding by the method of videography, a combination of ‘focused ethnography’ and ‘video analysis’ (Knoblauch and Schnettler 2012).

7. It is interesting to mention here that another project of the CRC *Affective Societies*, focusing on transnational emotional repertoires in reality TV-shows (‘Germany’s Next Topmodel’), found similar results. Lünenborg and Maier (2019) showed that their observed persons were expecting certain expressions *before* they were shown on screen. Of course, individual and collective forms differ from another, but the processes were the same: people do not follow an ‘inner’ basic emotion; they *expect* something to happen, even before it happens, as a form of sequential knowledge.
